# Foot orthoses for people with rheumatoid arthritis: a survey of prescription habits among podiatrists

**DOI:** 10.1186/s13047-019-0314-5

**Published:** 2019-01-25

**Authors:** Lara S. Chapman, Anthony C. Redmond, Karl B. Landorf, Keith Rome, Anne-Maree Keenan, Robin Waxman, Begonya Alcacer-Pitarch, Heidi J. Siddle, Michael R. Backhouse

**Affiliations:** 1 Department of Podiatry, Harrogate and District NHS Foundation Trust, Harrogate District Hospital, Lancaster Park Road, Harrogate, UK; 2 Leeds Institute of Rheumatic and Musculoskeletal Medicine, University of Leeds, Leeds, UK; 3 NIHR Leeds Biomedical Research Centre, Leeds Teaching Hospitals NHS Trust, Leeds, UK; 4 Discipline of Podiatry, School of Allied Health, La Trobe University, Melbourne, Australia; 5 La Trobe Sport and Exercise Medicine Research Centre, School of Allied Health, La Trobe University, Melbourne, Australia; 6 Health and Rehabilitation Research Institute and School of Podiatry, Auckland University of Technology, Auckland, New Zealand; 7 School of Healthcare, University of Leeds, Leeds, UK; 8 York Trials Unit, Department of Health Sciences, University of York, York, UK

**Keywords:** Foot, Orthotic devices, Orthoses, Rheumatoid arthritis, Podiatry

## Abstract

**Background:**

Guidelines recommend foot orthoses for people with both early (< 2 years) and established rheumatoid arthritis (RA). While prefabricated foot orthoses are cheaper and can exhibit comparable effects to customised devices, the available evidence for their effectiveness is inconsistent. Little is known about what types of foot orthoses clinicians prescribe. This study describes the foot orthoses prescription habits of podiatrists for people with rheumatoid arthritis.

**Methods:**

One hundred and eighty-three podiatrists from the United Kingdom (UK) (*n* = 88), Australia (*n* = 68) and New Zealand (*n* = 27) completed a self-administered, online survey regarding the types of foot orthoses prescribed in clinical practice for people with RA. This study forms part of a wider international survey exploring foot orthosis prescription habits.

**Results:**

UK respondents were more likely to prescribe prefabricated orthoses for early RA (*n* = 47, 53%) and customised orthoses for established RA (n = 47, 53%). Respondents in Australia were more likely to prescribe customised orthoses for both early (*n* = 32, 47%) and established (*n* = 46, 68%) RA, whilst respondents in New Zealand were more likely to prescribe prefabricated orthoses for both early (*n* = 16, 59%) and established (*n* = 10, 37%) disease.

Irrespective of disease stage, the use of foam impression boxes was more prevalent in the UK and New Zealand when capturing a model of the feet prior to manufacturing customised orthoses. In contrast, electronic scanning and plaster of Paris were more common in Australia. Computer aided manufacture was utilised more frequently among respondents in Australia than in the UK and New Zealand. Respondents in all three countries specified more flexible shell materials for established RA, compared to early disease. Cushioning top covers (e.g. PORON® or polyurethane) were most frequently specified in all countries for both disease stages.

**Conclusions:**

Considerable variation was seen in the self-reported foot orthoses prescription habits of respondents for people with RA. Variation between countries and disease stage was seen in type of orthoses, specific brands, manufacturing methods, and materials prescribed. The results allow podiatrists and broader health service providers to compare their practice against reported national and international patterns.

**Electronic supplementary material:**

The online version of this article (10.1186/s13047-019-0314-5) contains supplementary material, which is available to authorized users.

## Background

Foot orthoses (FOs) are frequently prescribed in clinical practice as an intervention for people with rheumatoid arthritis (RA), a chronic inflammatory disease with an estimated global prevalence of up to 1% [1]. The condition has significant economic impact. In 2009, RA accounted for over $355 million of Australian health expenditure [2], whilst a 2010 report estimated that the overall cost of productivity losses to the United Kingdom (UK) economy due to RA was almost £8 billion per year [3].

Foot pain is a prevalent and debilitating symptom of RA throughout the disease course, in both early (< 2 years) and established disease [4]. Foot pain frequently persists even when clinical remission of disease activity is achieved [5]. Studies consistently suggest that around 90% of people with RA experience foot problems during the course of their disease [4, 6]. Mechanical factors play a key role in the progression of foot deformity, and are increasingly thought to have a major role in the persistence of foot pathology [7–9]. Mechanical therapies, such as foot orthoses (FOs), offload painful joints and periarticular structures and are used to reduce pain, disability, and improve quality of life in people with RA [10, 11]. The foot is more amenable to treatment early in the disease course, prior to the development of irreversible joint damage and deformity [12, 13]. Earlier intervention with FOs has been linked to greater improvements in self-reported foot pain and disability [12]. As such, FOs are now widely recommended in key guidelines in the UK and Australia [14, 15].

FOs vary broadly in terms of their design, ranging from simple cushioning FOs to functional FOs, and their manufacturing methods, from generic mass produced prefabricated FOs to individual customised devices [16]. This variation is further confounded by additions such as posting, wedges and pads. Prefabricated FOs also vary considerably in terms of their physical form and material composition, which in turn affect their mechanical properties [17, 18].

Although systematic reviews have highlighted the need for more studies to determine the clinical and cost effectiveness of specific FOs in RA [19, 20], there are a number of small randomised controlled trials reported in the literature. Customised rigid and semi rigid FOs have been shown to reduce foot pain among people with RA who have metatarsalgia [21] and early rearfoot valgus [11]. However, the manufacture of custom FOs is complex and is frequently conducted by offsite commercial manufacturers often over a period of several weeks, which inherently delays the initiation of therapy, potentially reducing clinical benefit. Prefabricated FOs are, by definition, pre-made and can therefore be supplied immediately, eliminating the need to wait for off-site manufacture and enabling initiation of therapy as soon as the first clinical contact.

Some prefabricated FOs can exhibit comparable mechanical effects to more expensive custom devices [22, 23] and as such, prefabricated FOs may represent a substantial potential saving for health services [24]. Despite the frequency of foot complaints in RA and the financial burden of the condition on the economy, there is limited data relating to cost-effectiveness of FOs for people with RA [11, 25]. A recent exploratory study suggested that semi rigid customised FOs can improve pain and disability in people with established RA, compared to simple insoles. However, the customised FOs were more expensive to manufacture, with no significant cost per quality-adjusted life year gain [26]. An abundance of FO brands and sub-types are available on the market, but little is known about what types of FO are prescribed for people with RA, despite renewed interest in FO prescription patterns [27–30]. The only study to date to explore FO prescription habits for people with this condition was conducted over a decade ago [31]. The author reported that the majority of podiatrists surveyed prescribed non-rigid EVA FOs for people with early RA and simple accommodative FOs for people with established disease.

The aim of this study was to describe current FO prescription habits of podiatrists from the UK, Australia and New Zealand for people with RA.

## Methods

As part of a wider international survey of FO prescription habits among podiatrists, this descriptive study utilised a cross-sectional, online, self-administered survey to elicit FO prescription habits among registered podiatrists for people with RA. Ethical approval was received from the School of Medicine Research Ethics Committee, University of Leeds (Ref: MREC15–052). Subsequent approval was also gained from La Trobe University (Ref: MREC15–052) and Auckland University of Technology (Ref: 16/133). Participant consent was implied by completion of the survey and it was accessible from June 2016 to November 2016.

### Survey design

An electronic survey technique was used, utilising the Bristol Online Survey platform (http://onlinesurveys.ac.uk) to enable international completion. Further details of how the survey questions were developed and piloted are published elsewhere [30]. The survey (Additional file 1) contained a subsection designed to elicit FO prescription habits for people with early and established RA. In relation to each stage of the condition, respondents were asked to identify the type of FO most frequently prescribed, prefabricated FO brands provided, and the methods used to capture the 3D shape of the foot, manufacturing techniques, shell, rearfoot posting and top cover materials most frequently specified when prescribing customised FOs.

### Participants and data collection

Participants were invited to complete an anonymous online survey via professional e-newsletters, special interest groups, discussion forums, and professional publications, across the UK, Australia and New Zealand. Data from respondents practising in any other countries who participated in the survey were excluded from the main analysis due to potential differences in education and scope of practice, but were presented as supplementary data. The survey was also promoted at local and regional meetings during the study period. To be eligible to complete the survey, participants had to be registered podiatrists, able to access the survey online, and able to understand written English.

### Terminology

Within the survey, early RA referred to disease duration of 2 years or less, whilst established RA referred to those who had the condition for more than 2 years. In the absence of universally agreed definitions of FO types, FOs were described in three categories. Simple FOs were defined as flat insoles with or without padding to accommodate painful areas or lesions. Prefabricated FOs were considered as devices made to a generic foot shape, contoured for the arch, and included modular prefabricated orthoses that can be altered by clinicians (e.g. by the addition of posting, wedges, pads or top covers). Customised FOs were considered as devices manufactured for a specific person based on a 3D impression or computerised image of that person’s foot, and produced using computer aided device/manufacturing (CAD/CAM) or more traditional manufacturing techniques (e.g. foam impression box or plaster of Paris cast).

### Analysis

Survey data was entered into SPSS v 21 (Armonk, NY: IBM Corp) and analysed using descriptive statistics. Free text responses relating to prefabricated FOs prescribed were considered to be valid if they contained the name of a prefabricated FO brand. Invalid responses were excluded.

## Results

### Demographics

Two hundred and sixty-four (264) podiatrists completed the survey. The majority of respondents practised in the United Kingdom (47%, *n* = 124), Australia (30%, *n* = 79) and New Zealand (12%, *n* = 32).

Eighty-eight (71%) respondents in the UK indicated that they prescribed FOs for people with RA. The majority (*n* = 87, 99%) gained their primary podiatry qualification in the UK. Fifty-two (59%) UK respondents were female. UK respondents qualified between 1973 and 2016, with a mean (SD) of 19.7 (11.6) years since qualification. Thirty (34%) worked solely in the public sector, 29 (33%) worked solely in private practice, and 29 (33%) worked across both sectors. Comparisons between the public and private sectors were not made among respondents in Australia or New Zealand due to the limited number of respondents working solely in the public sector.

Sixty-eight (86%) respondents in Australia prescribed FOs for people with RA, 59 (87%) of whom qualified there. Thirty-four (50%) Australian respondents were female. Respondents in Australia qualified between 1968 and 2016, with a mean (SD) of 17.1 (11.5) years since qualification. Two (3%) Australian respondents worked solely in the public sector, 55 (81%) worked solely in private practice, and 11 (16%) worked across both sectors.

Twenty-seven (84%) respondents in New Zealand prescribed FOs for people with RA, 22 (82%) of whom qualified there. Eighteen (67%) New Zealand respondents were female. Respondents practising in New Zealand qualified between 1979 and 2016, with a mean (SD) of 15.14 (11.56) years since qualification. Two (7%) New Zealand respondents worked solely in the public sector, 10 (37%) worked solely in private practice, and 15 (56%) worked across both sectors.

Twenty-two (76%) respondents practising in other countries prescribed FOs for people with RA (Additional file 2).

### Types of FOs prescribed

**Fig. 1:**
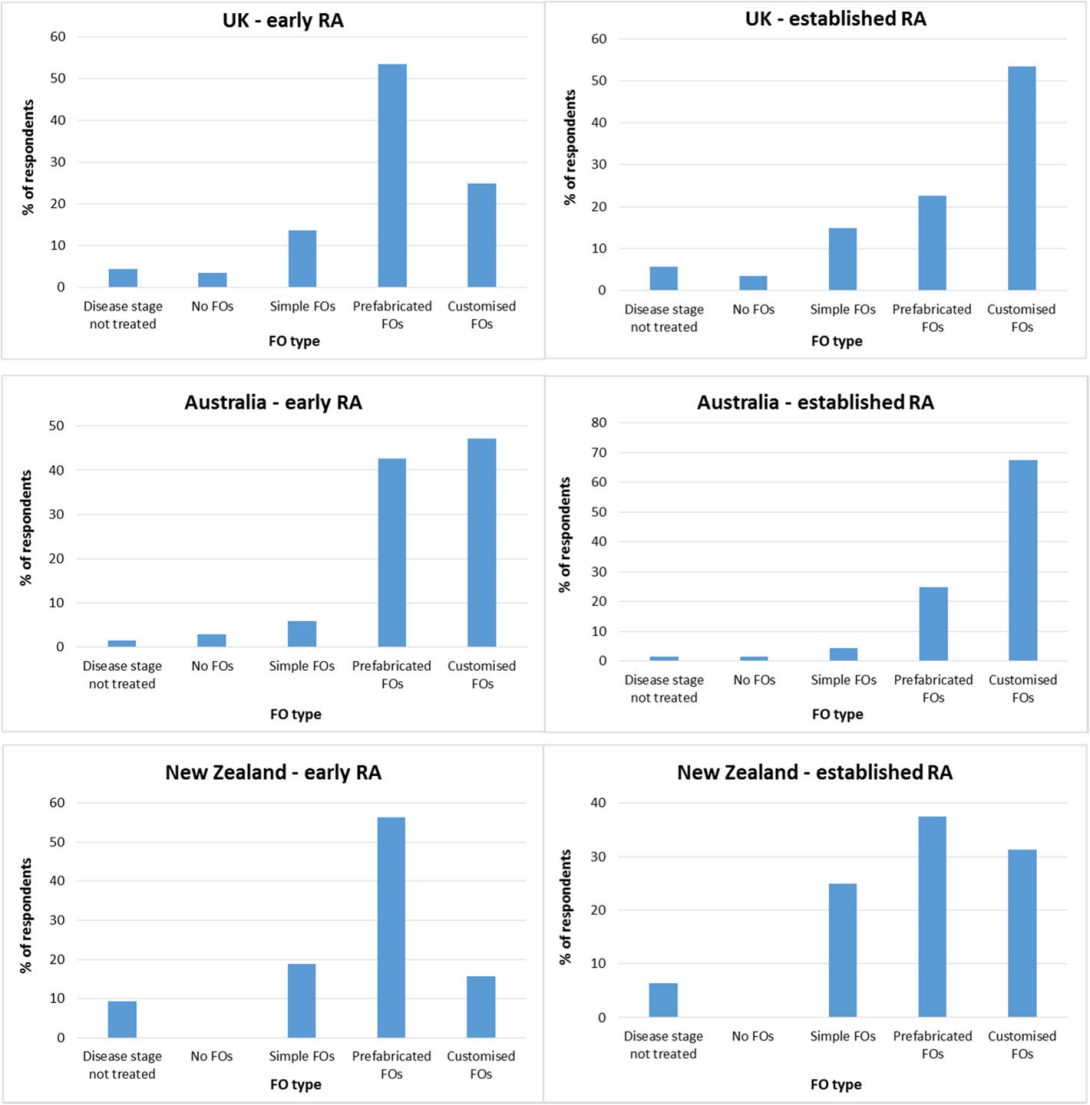
FO type most likely to be prescribed for early and established RA

Figure 1 shows the types of FOs respondents were most likely to prescribe for early and established RA. The majority of respondents in the UK and New Zealand reported they were more likely to prescribe prefabricated FOs than other FO types for early RA. Respondents in Australia reported they were more likely to prescribe customised FOs than other FO types for early RA, but were almost twice as likely to prescribe prefabricated FOs for early RA than for established RA.

**Fig. 2:**
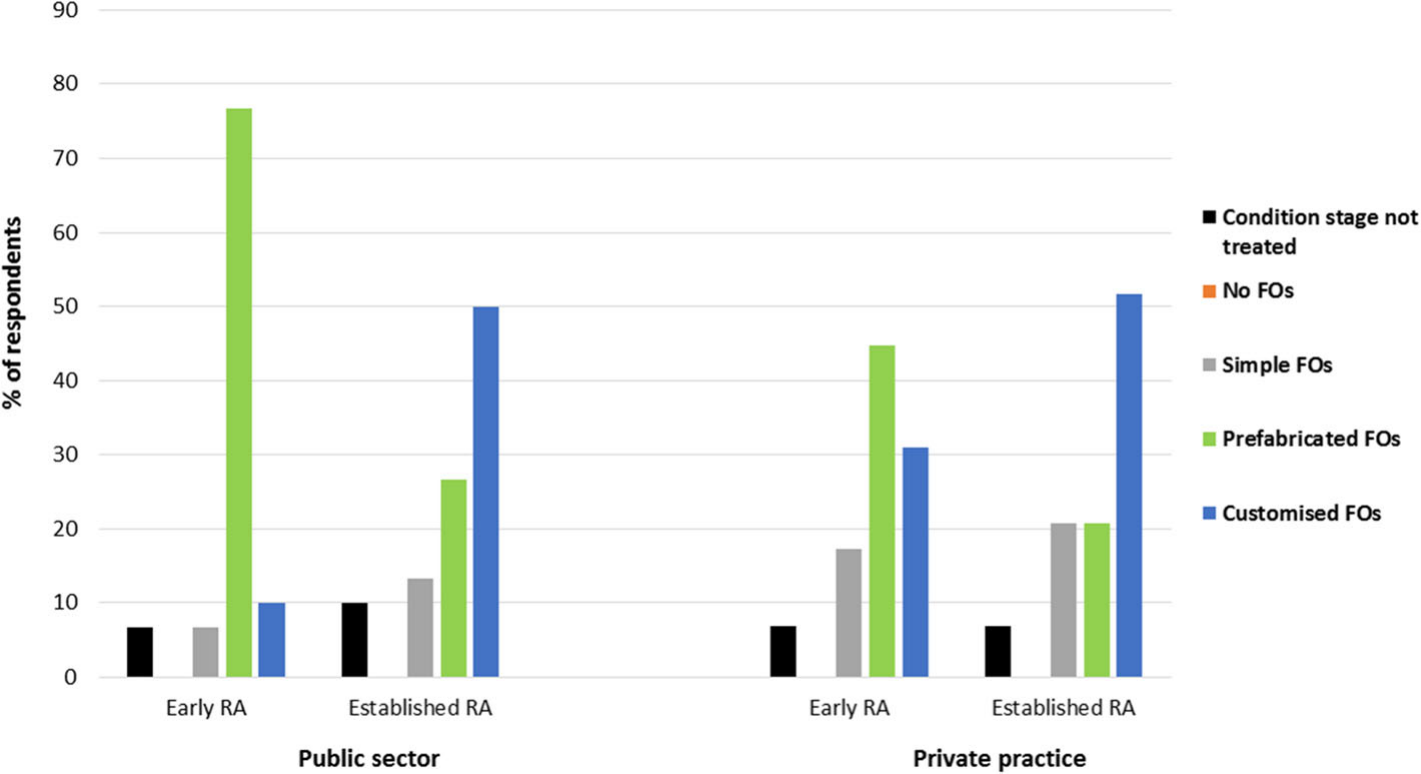
FO type prescribed for early and established RA among podiatrists in the UK by working sector

The majority of respondents in the UK and Australia reported they were more likely to prescribe customised FOs than other FO types for established RA. The prescription pattern among UK respondents was consistent regardless of whether they worked solely in the public sector or solely in private practice (Fig. 2). Respondents in New Zealand reported they were more likely to prescribe prefabricated FOs than other types of FO for established RA, but were twice as likely to prescribe customised FOs for established RA than for early RA.

### Prefabricated FOs

**Fig. 3:**
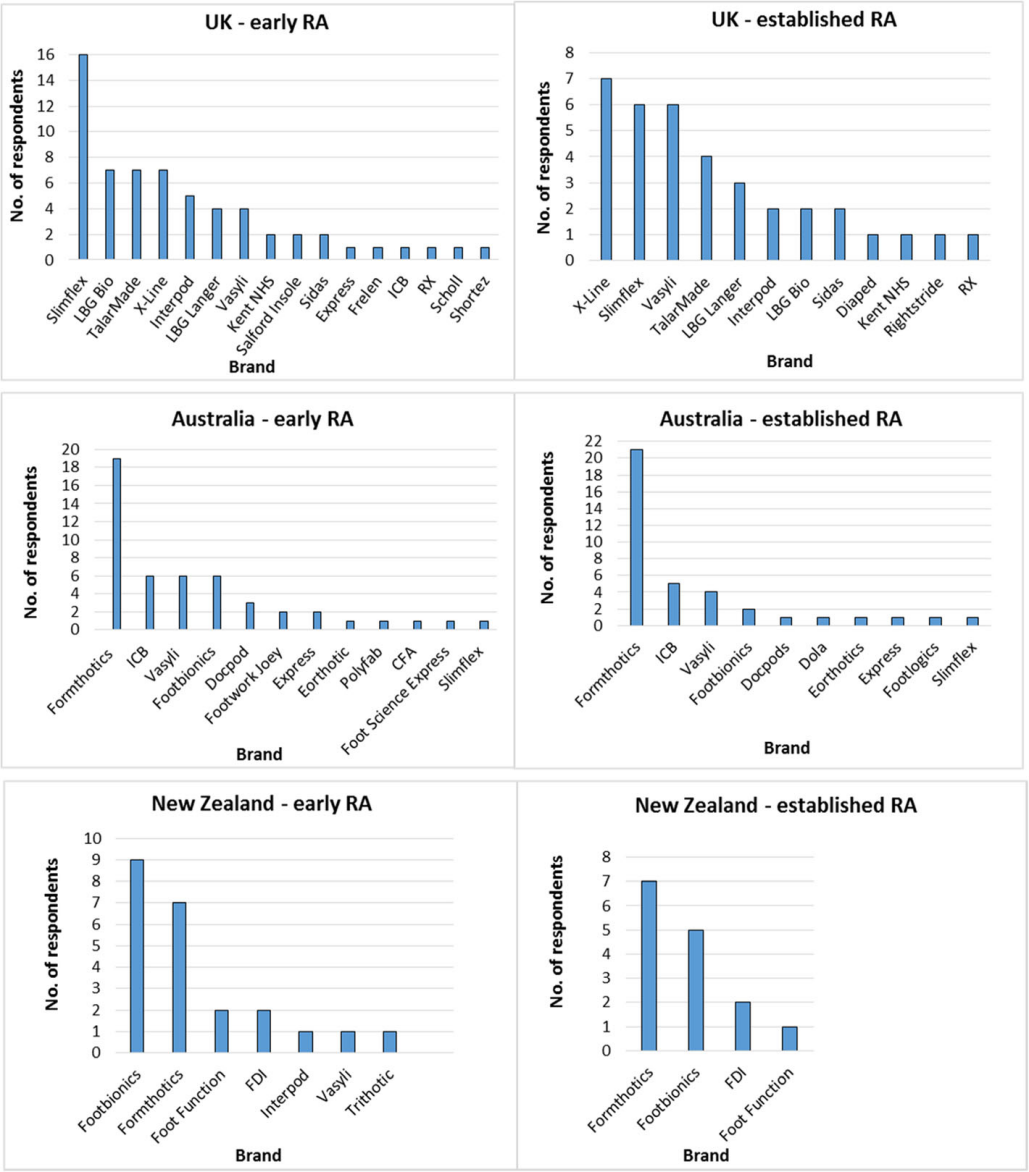
Most frequently prescribed prefabricated FO brands for early and established RA

Figure 3 illustrates the variety of prefabricated FO brands prescribed by respondents in clinical practice for early and established RA. Fifty-four (61%) UK respondents who prescribed FOs for RA in practice provided a valid response when asked which prefabricated FO was most frequently used for people with *early* RA. Of these, 47 (87%) indicated a single preferred brand, six (11%) indicated two preferred brands, and one (2%) respondent indicated three preferred brands. Sixteen different prefabricated FO brands were used among respondents for early RA. Slimflex® FOs were over twice as likely to be prescribed as other brands. However, when considering respondents working solely in private practice, Vasyli® and TalarMade™ were more frequently used (Table 1).

**Table 1:** Most frequently used prefabricated FO brands for early RA by sector

Brand	Solely public sector	Solely private practice	Combination of sectors	Total
Express	1	0	0	1
Frelen	0	1	0	1
ICB	1	0	0	1
Interpod	3	1	1	5
Kent NHS	0	1	1	2
LBG Bio	4	1	2	7
LBG Langer	0	3	1	4
Scholl	1	0	0	1
Salford Insole™	1	0	1	2
RX®	0	0	1	1
Shortez	1	0	0	1
Sidas	0	2	0	2
Slimflex®	10	1	5	16
TalarMade™	2	3	2	7
Vasyli®	0	4	0	4
X-Line®	4	0	3	7

Forty (59%) respondents in Australia provided a valid response when asked which prefabricated FO was most frequently used for people with early RA. Thirty-four (85%) indicated a single preferred brand, five (12.5%) indicated two preferred brands, and one (2.5%) respondent indicated three preferred brands. Twelve different prefabricated FO brands were used among respondents for early RA. Formthotics™ were over three times more likely to be prescribed by respondents in Australia for early RA than other prefabricated FO brands.

Twenty-one (78%) respondents in New Zealand provided a valid response when asked which prefabricated FO was most frequently used for people with early RA. Nineteen (90%) indicated a single preferred brand, whilst two (10%) respondents indicated two preferred brands. Seven different prefabricated FO brands were used among respondents for early RA. Footbionics® was the most frequently specified brand.

Twenty-eight (32%) UK respondents provided a valid response when asked which prefabricated FO they most frequently used for people with *established* RA. Twenty-three (82%) stated one preferred prefabricated FO brand, four (14%) stated two brands, and one (4%) respondent stated five brands. Twelve different prefabricated FO brands were used among UK respondents for established RA. Overall, X-Line® was the most frequently prescribed brand, followed by Slimflex® and Vasyli®. However, none of the UK respondents working solely in private practice indicated Slimflex® or X-Line® as a preferred brand (Table 2).

**Table 2:** Most frequently used prefabricated FO brands for established RA by sector

Brand	Solely public sector	Solely private practice	Combination of sectors	Total
Diaped	0	0	1	1
Interpod	1	0	1	2
Kent NHS	0	0	1	1
LBG Bio	1	1	0	2
LBG Langer	0	1	2	3
Rightstride®	1	0	0	1
RX®	0	0	1	1
Sidas	0	2	0	2
Slimflex®	4	0	2	6
TalarMade™	1	0	3	4
Vasyli®	0	4	2	6
X-Line®	4	0	3	7

Thirty-eight (56%) Australian respondents provided a valid response when asked which prefabricated FO they most frequently used for people with established RA. Thirty-five (92%) indicated a single preferred brand, two (5%) indicated two preferred brands, and one (3%) respondent indicated three preferred brands. Ten different prefabricated FO brands were used among respondents in Australia for established RA. Formthotics™ were over four times more likely to be prescribed than other prefabricated FO brands.

Thirteen (48%) respondents in New Zealand gave a valid response when asked which prefabricated FO they most frequently used for people with established RA. Eleven (85%) indicated one preferred prefabricated FO brand, and two (15%) respondents indicated two preferred brands. Four different prefabricated FO brands were used among respondents in New Zealand for established RA; Formthotics™ was the most frequently specified brand.

### Customised FO provision

Forty-seven (53%) respondents in the UK indicated that they prescribed customised FOs for people with early RA at least some of the time, compared to 48 (71%) respondents in Australia and 16 (59%) in New Zealand. For established RA, 63 (72%) UK respondents indicated that they prescribed customised FOs at least some of the time, compared to 59 (87%) respondents in Australia and 18 (67%) in New Zealand.

### Manufacturing methods

Table 3 illustrates the range of manufacturing methods and materials specified when prescribing customised FOs for early and established RA. Use of foam impression boxes was most frequently reported among respondents in the UK to capture the 3D shape of the foot in both disease stages, regardless of working sector (Table 4). Respondents in New Zealand also reported using foam impression boxes most frequently for both stages of RA. In contrast, respondents in Australia reported using foam impression boxes least frequently, with almost equal use of plaster of Paris and electronic scanning. Comparisons between the public sector and private practice were not made among respondents in Australia or New Zealand due to the limited number of respondents working solely in the public sector.

**Table 3:** Customised FO prescription habits

	UK		Australia		New Zealand	
	Early RA (*n* = 47)	Established RA (*n* = 63)	Early RA (*n* = 48)	Established RA (*n* = 59)	Early RA (*n* = 16)	Established RA (*n* = 18)
Methods used to capture 3D shape of foot						
Plaster of Paris	13 (28%)	17 (27%)	21 (44%)	25 (42%)	5 (31%)	6 (33%)
Foam impression box	28 (59%)	40 (63.5%)	7 (14%)	10 (17%)	9 (56%)	11 (61%)
Electronic scanning/ imaging	6 (13%)	6 (9.5%)	20 (42%)	24 (41%)	2 (13%)	1 (6%)
Weightbearing	21 (45%)	33 (52%)	9 (19%)	16 (27%)	7 (44%)	10 (56%)
Non-weightbearing	26 (55%)	30 (48%)	39 (81%)	43 (73%)	9 (56%)	8 (44%)
Manufacturing techniques						
Computer aided manufacture	20 (43%)	30 (48%)	38 (79%)	44 (75%)	8 (50%)	8 (44%)
Traditional manufacturing techniques	27 (57%)	33 (52%)	10 (21%)	15 (25%)	8 (50%)	10 (56%)
Shell material						
Highly rigid	6 (13%)	1 (2%)	1 (2%)	1 (2%)	0 (0%)	0 (0%)
Semi rigid	17 (36%)	13 (21%)	20 (42%)	16 (27%)	6 (38%)	3 (17%)
Semi flexible	17 (36%)	28 (44%)	17 (35%)	25 (42%)	9 (56%)	5 (27%)
Highly flexible	7 (5%)	21 (33%)	10 (21%)	17 (29%)	1 (6%)	10 (56%)
Rearfoot posting material						
None	6 (13%)	9 (14%)	2 (4%)	4 (7%)	0 (0%)	2 (11%)
Intrinsic	15 (32%)	17 (27%)	12 (25%)	15 (25%)	2 (12.5%)	4 (22%)
Highly rigid	0 (0%)	0 (0%)	1 (2%)	1 (2%)	0 (0%)	0 (0%)
Semi rigid	5 (10%)	3 (5%)	4 (8%)	4 (7%)	1 (6%)	1 (6%)
Semi flexible	21 (45%)	27 (43%)	23 (48%)	27 (46%)	11 (69%)	3 (17%)
Highly flexible	0 (0%)	7 (11%)	6 (13%)	8 (13%)	2 (12.5%)	8 (44%)
Top cover ^a^						
Minimal	9	6	3	3	1	0
Cushioning	24	30	26	25	11	11
Cushioning with modification to forefoot	21	41	29	45	7	12
Cushioning with modification to midfoot	11	18	10	18	5	7
Cushioning with modification to rearfoot	8	16	7	14	5	6

^a^ Respondents could select more than one choice for most frequently specified top cover

**Table 4:** Customised FO prescription habits by UK sector

	Early RA (*n* = 47)			Established RA (*n* = 63)		
	Solely public sector (*n* = 12)	Solely private practice (*n* = 18)	Combination (*n* = 17)	Solely public sector (*n* = 19)	Solely private practice (*n* = 21)	Combination (*n* = 23)
Methods used to capture 3D shape of foot						
Plaster of Paris	1 (8.3%)	6 (33.3%)	6 (35.3%)	2 (10.5%)	7 (33.3%)	8 (34.8%)
Foam impression box	11 (91.7%)	9 (50%)	8 (47.1%)	17 (89.5%)	11 (52.4%)	12 (52.2%)
Electronic scanning/ imaging	0 (0%)	3 (16.7%)	3 (17.6%)	0 (0%)	3 (14.3%)	3 (13.0%)
Weightbearing	7 (58.3%)	8 (44.4%)	6 (35.3%)	11 (57.9%)	9 (42.9%)	13 (56.5%)
Non-weightbearing	5 (41.7%)	10 (55.6%)	11 (64.7%)	8 (42.1%)	12 (57.1%)	10 (43.5%)
Manufacturing techniques						
Computer aided manufacture	3 (25%)	9 (50%)	8 (47.1%)	9 (47.4%)	12 (57.1%)	9 (39.1%)
Traditional manufacturing techniques	9 (75%)	9 (50%)	9 (52.9%)	10 (52.6%)	9 (42.9%)	14 (60.9%)
Shell material						
Highly rigid	1 (8.3%)	2 (11.1%)	3 (17.6%)	0 (0.0%)	0 (0.0%)	1 (4.3%)
Semi rigid	4 (33.3%)	5 (27.8%)	8 (47.1%)	2 (10.5%)	7 (33.3%)	4 (17.4%)
Semi flexible	5 (41.7%)	8 (44.4%)	4 (23.5%)	8 (42.1%)	8 (38.1%)	12 (52.2%)
Highly flexible	2 (16.7%)	3 (16.7%)	2 (11.8%)	9 (47.4%)	6 (28.6%)	6 (26.1%)
Rearfoot posting material						
None	3 (25%)	2 (11.1%)	1 (5.9%)	3 (15.8%)	3 (14.3%)	3 (13.0%)
Intrinsic	4 (33.3%)	6 (33.3%)	5 (29.4%)	5 (26.3%)	6 (28.6%)	6 (26.1%)
Highly rigid	0 (0.0%)	0 (0.0%)	0 (0.0%)	0 (0.0%)	0 (0.0%)	0 (0.0%)
Semi rigid	1 (8.3%)	1 (5.6%)	3 (17.6%)	0 (0.0%)	1 (4.8%)	2 (8.7%)
Semi flexible	4 (33.3%)	9 (50%)	8 (47.1%)	10 (52.6%)	9 (42.9%)	8 (34.8%)
Highly flexible	0 (0.0%)	0 (0.0%)	0 (0.0%)	1 (5.3%)	2 (9.5%)	4 (17.4%)
Top cover						
Minimal	4	3	2	2	3	1
Cushioning	6	10	8	11	9	10
Cushioning with modification to forefoot	6	9	6	12	15	14
Cushioning with modification to midfoot	3	5	3	8	6	4
Cushioning with modification to rearfoot	3	4	1	6	6	4

Respondents in the UK and New Zealand reported they were slightly more likely to use non-weightbearing methods to capture the 3D shape of the foot in early RA, and weightbearing methods in established RA. A large majority of respondents in Australia reported using non-weightbearing methods for both stages of RA.

UK respondents reported they were slightly more likely to use traditional manufacturing techniques (e.g. vacuum forming), as opposed to computer aided manufacture, for both stages of RA. Reported use of computer aided manufacture was higher among respondents working solely in UK private practice than those working solely in the public sector (Table 4). Respondents in New Zealand also reported they were slightly more likely to use traditional manufacturing techniques for early RA, although use of traditional and computer aided manufacture was equal for established RA. Respondents in Australia reported they were three times more likely to use computer aided FO manufacture than traditional manufacture for early RA and over twice as likely for established RA.

### Materials

For early RA, UK respondents most frequently reported specifying semi flexible (e.g. high density EVA) and semi rigid (e.g. polypropylene) customised FO shell materials. Semi rigid shell materials were most frequently specified for early RA among Australian respondents, whereas New Zealand respondents most frequently specified semi flexible materials. Respondents in all three countries reported most frequently specifying semiflexible rearfoot posting materials in early RA. For established RA, semi flexible shell and rearfoot materials were reported to be most frequently specified by UK and Australian respondents. Comparatively, New Zealand respondents reported most frequently specifying highly flexible (e.g. medium or low density EVA) shell and rearfoot posting materials. Respondents in the UK and New Zealand reported most frequently specifying cushioning (e.g. PORON®) as a top cover for early RA, and cushioning with specific modification or offloading to the forefoot for established RA. Cushioning with specific modification or offloading to the forefoot was the most reported frequently specified top cover among respondents in Australia for both stages of the condition.

## Discussion

This study identified the types of prefabricated FOs used by respondents in contemporary clinical practice for the treatment of early and established RA, allowing podiatrists and broader health service providers to compare their practice against reported national and international prescription habits.

Our findings indicate that there is variation across countries and between sectors in the types of FO prescribed. The majority of respondents in the UK, Australia and New Zealand reported prescribing FOs for RA, in line with current guidelines [14]. In the UK, respondents reported they would be more likely to prescribe prefabricated FOs for early RA and customised FOs for established RA. Respondents in Australia reported they were more likely to prescribe customised FOs for both stages of the condition, whilst those in New Zealand reported were more likely to prescribe prefabricated FOs for both stages. These variations in prescription habits between countries may reflect different health systems and the subsequent health insurance schemes in place, but further work is required to explore this fully.

Interestingly, respondents in Australia reported they were twice as likely to prescribe prefabricated FOs for early RA compared to established disease, and those in New Zealand reported they were twice as likely to prescribe customised FOs for established disease compared to early RA. Reported customised FO shell material prescriptions also differed according to disease stage. There is lack of consensus within the podiatry profession across all three countries [27, 30] regarding which type of FO should be prescribed for specific conditions, and the materials to use in FO prescriptions. There is also an absence of current guidelines to support these decisions. Our results suggest that respondents may stratify FO prescriptions for people with RA based on the stage of the condition, but further work is needed to explore clinical decision making.

Our results support a previous exploratory survey of FO prescription habits for people with RA [31], in which the majority of UK respondents prescribed high or medium density EVA FOs for early RA. However, our findings indicate that customised FOs were reported as most frequently prescribed by UK respondents for people with established RA, whereas the former survey found that soft accommodative FOs were prescribed most often for this stage of the condition. This difference in prescription habits may reflect the development of national guidelines in the decade since the previous survey was conducted, with the provision of functional FOs now recommended [14].

Our study is the first of its kind to differentiate the types of prefabricated FOs prescribed in clinical practice for the treatment of early and established RA. A range of prefabricated FO brands and models were prescribed by respondents in practice. Our study found that Slimflex® and X-Line® brands were most commonly prescribed in the UK. Comparatively, Formthotics™ was the most commonly prescribed brand in Australia, and Formthotics™ and Footbionics*®* were the most commonly prescribed brands in New Zealand, despite a current lack of evidence suggesting that any specific prefabricated FO is more effective than any other. Nevertheless, this most likely reflects which prefabricated FOs are most commonly used for all foot problems in these countries.

Irrespective of disease stage and working sector, we found that foam impression boxes were most frequently used among UK respondents to capture the shape of the foot when manufacturing customised FOs. Respondents in New Zealand exhibited similar habits for obtaining an impression of the feet for customised FO. Although our results are not directly comparable, these findings are similar to those from the recent study by Nester et al., where 54% of the podiatrists, physiotherapists and orthotists surveyed in the UK used foam impression boxes to capture foot shape, 14% used a scanner, and manual manufacture and computer aided manufacture were used in almost equal measure [29]. However, our sample consisted entirely of podiatrists, therefore results should not be extrapolated to other professions. Three-dimensional (3D) scanning is considered to be more reproducible than foam impression boxes and plaster of Paris in capturing the shape of the foot [32]. However, barriers to the use of technology in clinical practice, including usability issues and lack of training, have previously been identified among UK practitioners prescribing customised FOs within the NHS [33]. Our study found higher use of electronic scanning among UK respondents working solely in private practice compared to those working solely in the public sector, with none of the latter group using this method for either stage of the condition. Electronic scanning and computer aided manufacture were used more frequently among respondents in Australia compared to the UK and New Zealand.

Semi flexible rearfoot posting materials (e.g. high density EVA) were most frequently specified for customised FOs for both disease stages in the UK and Australia in our study, whilst cushioning (e.g. PORON®) top cover materials were most frequently specified in all three countries. These findings concur with published data relating to FO prescription habits unspecific to RA, from over a decade ago [27], different countries [27, 28] and professions outside of podiatry [29]. However, our study found that rearfoot posting materials for customised FOs differed among respondents in New Zealand between the two disease stages. Customised FO shell material specifications also varied according to the stage of RA in all three countries. Results relating to shell materials among respondents in the UK and Australia were inconsistent with previous studies investigating general customised FO prescription habits, where respondents were more likely to specify a polypropylene or medium density EVA shell [27–29].

There are several limitations that need to be acknowledged when considering the findings of this study. The open invitation method of survey distribution did not allow a denominator population of podiatrists to be determined, therefore it was not possible to estimate a response rate. As there are approximately 4800 registered podiatrists in Australia, 12,700 in the UK and 450 in New Zealand, the generalisability of our findings may be limited. However, our findings are largely similar to previous published data and the survey did elicit detailed information about FO prescription habits for RA, leading to a compromise between the depth of information and breadth of population covered.

Secondly, there were inconsistencies in responses from two participants, who in an earlier stage of the survey stated they did not treat either stage of the condition in practice, but in the RA section, stated they did and provided detailed responses relating to their FO prescription habits for people with RA. This data was included in the analysis, but suggests the results should be interpreted with this in mind.

Thirdly, although specific definitions of simple FOs, prefabricated FOs and customised FOs were provided at multiple points within the survey, it is possible that some participants misunderstood, which has implications for the reliability of data relating to the type of FO prescribed for each stage of the condition. For example, when asked to specify the prefabricated FO brand most frequently prescribed for each stage of the disease, several respondents from each country gave invalid responses including descriptions of customised and simple FOs (Additional file 3).

Additionally, as with any survey, there is a potential for responder bias; respondents may have been those with an interest in foot orthoses, and it cannot be established whether the responses of those who did not complete the survey would have differed. The study asked respondents to identify what FO prescriptions they most frequently specified, therefore the potential for recall bias is always a consideration with this methodology. However, the electronic survey technique allowed for a wide breadth of clinical and geographical coverage, and aimed to reduce the burden placed on respondents.

Finally, the wording of surveys is difficult at the best of times, and variations in terminology may affect a survey conducted online across multiple countries. For example, a term such as ‘semi rigid’ as it relates to orthotic therapy may mean different things to different practitioners. Nevertheless, we believe that we obtained a reasonable balance by using terminology and providing explanations that would have been understood by the majority of respondents, although this is an issue that requires careful consideration for similar surveys in the future.

Future research is needed to explore the clinical reasoning behind FO prescription choices. Several participants indicated in free text additional comments at the end of the survey that their FO prescriptions for RA would depend on individual presentations (Additional file 3). This included assessment findings, such as joint range of motion, foot shape and deformity, and subjective symptoms, such as the presence and location of pain. These comments reflect previous research suggesting that practitioners are influenced by patient history, foot type, and the aim of treatment when prescribing FOs [33]. Budget constraints, with regards to the clinician, service provider and individual patient, were also identified by respondents in the current survey as factors that affect decisions relating to FO prescriptions.

This study identified the types of prefabricated FOs used in contemporary clinical practice for the treatment of early and established RA, allowing podiatrists and broader health service providers to compare their practice against reported national and international prescription habits. Given the frequency of foot complaints in RA [4, 6] and the potential cost savings to health services from using prefabricated FOs as opposed to customised devices [24], there is a clear indication of a need for further research into the clinical and cost effectiveness of prefabricated FOs for RA. The need for more definitive randomised controlled trials for customised FOs in the management of RA has also been identified in the literature [19, 20]. To date, clinical trials have often selected FOs based on investigator preference rather than specific properties of the device, mechanical effects, or how widely used they are in clinical practice. Furthermore, the choice of materials and design of customised FOs used in RA trials has not always been reported, potentially limiting the translation of trial findings to clinical practice. Findings from our study can inform future research investigating the physical properties of specific prefabricated and customised FOs for RA, and their clinical and cost effectiveness, ensuring these studies are relevant to clinical practice.

## Conclusions

This study describes the most common FOs prescriptions for people with RA within clinical practice in the UK, Australia and New Zealand. Findings indicated variation across countries and between disease stages in the types of orthoses prescribed, although the majority of respondents in the UK, Australia and New Zealand prescribed functional insoles for RA, in line with current guidelines. Variation was seen in the specific brands of prefabricated FOs prescribed, and in the manufacturing methods and materials used when prescribing customised FOs. There is currently a lack of evidence to inform prescription choices and this should be addressed through future research. The results allow podiatrists and broader health service providers to compare their practice against reported national and international patterns, and inform future research investigating the effectiveness of specific prefabricated and customised FOs for people with RA.

## Additional files

Additional file 1: Foot orthoses survey full. (PDF 119 kb)

Additional file 2: Supplementary data from other countries. (DOCX 16 kb)

Additional file 3: Free text comments. (DOCX 13 kb)

## Abbreviations

EVA: Ethyl vinyl acetate
FOs: Foot orthoses
NHS: National Health Service
RA: Rheumatoid arthritis
UK: United Kingdom
