# Supplementary material for: Foot orthoses for people with rheumatoid arthritis: a survey of prescription habits among podiatrists
Source: J Foot Ankle Res. 2019 Jan 25;12:7. doi: 10.1186/s13047-019-0314-5 (PMC6347791; doi:10.1186/s13047-019-0314-5)
Supplement: Supplementary file 2 — Supplementary data from other countries. (DOCX 16 kb) [file 13047_2019_314_MOESM2_ESM.docx]

**Additional file 2: Supplementary data (other countries)**

Table 5 displays the countries of practice of the podiatrists practising outside of the UK, Australia and New Zealand who prescribed FOs for people with RA. Of these podiatrists, 14 (64%) were male. Podiatrists qualified between 1976 and 2015, with a mean (SD) of 16.5 (13.16) years since qualification. Three (14%) podiatrists worked solely in the public sector, 12 (54%) worked solely in the public sector, and 7 (32%) worked across both sectors.

**Table 5** Overview of podiatrists’ countries of practice

| **Country of practice** | **No. of podiatrists (n = 22)** |
| --- | --- |
| Republic of Ireland | 8 |
| Canada | 3 |
| South Africa | 3 |
| United States | 2 |
| Singapore | 2 |
| Hong Kong | 1 |
| Italy/France/Spain | 1 |
| Barbados | 1 |
| Romania | 1 |

*Types of FOs prescribed*

When asked about early RA, three podiatrists (14%) stated that they did not prescribe FOs for this disease stage, one (4%) was most likely to prescribe simple FOs, nine (41%) were most likely to prescribe prefabricated FOs, and nine (41%) were most likely to prescribe customised FOs. For established RA, two (9%) podiatrists stated that they did not prescribe FOs for this disease stage, two (9%) were most likely to prescribe prefabricated FOs, and 18 (82%) were most likely to prescribe customised FOs.

*Prefabricated FOs*

Nine podiatrists revealed the prefabricated FO brands they prescribed most frequently for early RA, whilst six podiatrists indicated the brands that they prescribed most frequently for established RA. The range of brands are shown in Table 6.

**Table 6** Range of prefabricated FO brands prescribed for early and established RA amongst podiatrists from other countries

| **Prefabricated FO brands (early RA)** | **Prefabricated FO brands (established RA)** |
| --- | --- |
| Alimed | Formthotics™ |
| FL | ICS |
| Formthotics™ | Oolab |
| Oolab | PPL |
| PPL | TalarMade™ |
| TalarMade™ | X-Line® |
| Vasyli® |  |
| X-Line® |  |

*Customised FO provision*

Thirteen (59%) podiatrists indicated that they prescribed customised FOs for patients with early RA, at least some of the time, increasing to 20 (91%) podiatrists for established RA. Prescription habits relating to customised FOs are displayed in Table 7.

**Table 7** Customised FO prescription habits amongst podiatrists from other countries

|  | **Early RA (n=13)** | **Established RA (n=20)** |
| --- | --- | --- |
| **Methods used to capture 3D shape of foot** |  |  |
| Plaster of Paris | 6 (46%) | 9 (45%) |
| Foam impression box | 2 (15%) | 5 (25%) |
| Electric scanning/ imaging | 5 (39%) | 6 (30%) |
| Weightbearing | 4 (31%) | 5 (25%) |
| Non-weightbearing | 9 (69%) | 15 (75%) |
| **Manufacturing techniques** |  |  |
| Computer aided manufacture | 6 (46%) | 6 (30%) |
| Traditional manufacturing techniques | 7 (54%) | 14 (70%) |
| **Shell material** |  |  |
| Highly rigid | 0 (0%) | 0 (0%) |
| Semi rigid | 6 (46%) | 4 (20%) |
| Semi flexible | 6 (46%) | 11 (55%) |
| Highly flexible | 1 (8%) | 5 (25%) |
| **Rearfoot posting material** |  |  |
| None | 1 (8%) | 2 (10%) |
| Intrinsic | 3 (23%) | 4 (20%) |
| Highly rigid | 0 (0%) | 0 (0%) |
| Semi rigid | 1 (8%) | 1 (5%) |
| Semi flexible | 6 (46%) | 10 (50%) |
| Highly flexible | 2 (15%) | 3 (15%) |
| **Top cover** |  |  |
| Minimal | 1 | 0 |
| Cushioning | 8 | 10 |
| Cushioning with modification to forefoot | 4 | 9 |
| Cushioning with modification to midfoot | 2 | 6 |
| Cushioning with modification to rearfoot | 1 | 3 |
