# Supplementary material for: Foot orthoses for people with rheumatoid arthritis: a survey of prescription habits among podiatrists
Source: J Foot Ankle Res. 2019 Jan 25;12:7. doi: 10.1186/s13047-019-0314-5 (PMC6347791; doi:10.1186/s13047-019-0314-5)
Supplement: Supplementary file 3 — Free text comments. (DOCX 13 kb) [file 13047_2019_314_MOESM3_ESM.docx]

| The decision on whether to prescribe prefabricated or custom devices is made case by case. The two key factors are pain and deformity. Mild pain or deformity typically warrants a prefabricated orthotic. More intense / chronic pain or more advance deformity warrants custom. The patients budget, life |
| --- |
| A number of the questions could have multiple answers, so made it difficult to give a comprehensive answer and a true reflection of my practice.(E.g The established RA patient was not identified as having deformity or pain or both. This would influence the orthotic choice) |
| Have stopped using orthotics for MSK conditions - use manual therapy and exercise |
| I am new to this job and I haven't done a lot of insole prescribing. Wasn't a big priority in my induction. |
| I do not routinely see patient with RhA though there are a small number that I would see in my MSK foot and ankle clinic. |
| I find Vasyli products generally the most versatile product but for more dynamic foot conditions I find simple insoles or cradle orthotics more appropriate. |
| I found the questions reductive and did not capture the scope of presentations of particular diseases, nor my treatment |
| I mainly use custom orthoses for children and sports patients who tolerate a prefab but need a more durable orthotic. |
| I refer into the NHS service for customised FOs for rheumatoid patients and diabetic neuropathy with pathology |
| I tend to refer patients to NHS if they have complex conditions |
| I use a low level photodynamic laser for the treatment of inflammatory arthritis |
| I usually manufacture my own insoles due to cost and convienence. |
| I will be interested to read the results |
| I work in an NHS Trust that does not allow me to use casted foot orthoses due to budget constraints. Therefore all my answers are negative for casted orthoses. |
| Just to add that where I do not prescribe bespoke orthoses I will refer patients that require them onto colleagues that do |
| Most of the answers are subjective to the requirements of the patient and their symptoms. |
| Options with diabetes neuropathy and non-neuropathy are vague, could be treating anything |
| Primarily treat MSK conditions with manual therapy |
| Regarding your questions about the types of orthotics used to treat different conditions, I don't only use a certain orthotic with a certain condition. I will use the orthotic that best controls that individual based the findings of my assessment, e.g. if they have more than 6 degrees difference |
| Regularly refer on for customised orthosis |
| The questions do not allow for multiple types of FOs to treat for the same condition which limits the accuracy of my data |
| This survery did not give any options to show where discretion is used depending on the presentation of the patient. |
| This survey has been very thought provoking & somewhat challenging! I have generalised my answers as accurately as possible to reflect my prescribing habits. So many variables affect what I prescribe if any, eg footwear, complexity of pt presentation, likely compliance, cost etc... |
| We have developed a silicone combination orthotic that reduces forces from 27kg-1kg/sqcm |
